# Lobster Yield Dynamics in a Warming Ocean: A Generalized Linear Modeling Case Study in Prince Edward Island, Canada

**DOI:** 10.3390/foods14122072

**Published:** 2025-06-12

**Authors:** Manzura Khan, Xiuquan Wang, Krishna Kumar Thakur, Ryan Guild, Rana Ali Nawaz, Muhammad Awais

**Affiliations:** 1 Canadian Centre for Climate Change and Adaptation, University of Prince Edward Island, St. Peter’s Bay, PE C0A 2A0, Canada; manzura_poly@yahoo.co.uk (M.K.);; 2 School of Climate Change and Adaptation, University of Prince Edward Island, Charlottetown, PE C1A 4P3, Canada; 3 Atlantic Veterinary College, University of Prince Edward Island, Charlottetown, PE C1A 4P3, Canada; kthakur@upei.ca

**Keywords:** lobster, climate change, sea surface temperature, future projection

## Abstract

The lobster fishery is the third largest industry in Prince Edward Island (PEI), Atlantic Canada. Rising water temperatures due to global warming are impacting the successful completion of the lobster life cycle, which is heavily dependent on water temperature. This study investigated the relationship between lobster landings and sea surface temperature (SST) in PEI. Using Generalized Linear Models (GLM), we identified a significant correlation between annual historical lobster landings and monthly sea surface temperatures (SST) in the waters around PEI from 1990 to 2021. Considering the 5–8 year maturation period of lobsters, we applied a lagged SST structure over an 8-year period and used a Generalized Linear Model (GLM) to evaluate the relationship between historical SST and lobster landings. Our findings suggest that historical increases in SST are correlated with changes in lobster landings. Given the known sensitivities of lobster life cycles (i.e., spawning, larval development) and behavior (i.e., mating) to high ambient water temperature, our study also offers important insights for future fishery management under anticipated climate change scenarios.

## 1. Introduction

Lobster is an important fisheries resource in Prince Edward Island. The lobster industry, valued at CAD $438 million and employing over 8500 people, is the third largest in PEI, with more than 1200 licensed fishers actively engaged across its three main Lobster Fishing Areas (LFAs) [1]. Over the past few decades, the lobster fishery has grown to represent a considerable portion of marine harvests in Canada [2]. For example, lobster fisheries accounted for 69.7% and 58.5% of all fishery landings (i.e., yield) in PEI in 2017 and 2020, respectively [3]. PEI fishers represent approximately 13% of the over 9000 Canadian fishers and 9% of over 13,000 North American fishers [4], emphasizing the magnitude of this sector for the economy of PEI. The American lobster (Homarus americanus), commonly referred to as lobster, is distributed across the continental shelf of the Northwest Atlantic, ranging from Newfoundland, Canada, to the southern coast of North Carolina, USA [5]. Lobsters are mainly found in waters less than 50 m in depth [6], but they can also be found in waters up to 700 m deep [7]. Since lobsters are ectothermic animals, ambient temperature has a significant impact on their physiological functions, including growth, reproduction, metabolism, and survival [8,9,10]. Adult lobsters exhibit a wide temperature tolerance, surviving in waters ranging from −1 °C to 30 °C [11]. However, their larval stages are considerably more sensitive to temperature fluctuations [12,13]. While some studies suggest faster larval growth in warmer waters within a suitable range [2], exceeding this range can have detrimental effects on lobster larvae. Research suggests that high temperatures can disrupt larval development, survival, and recruitment [14]. Post-larval lobsters show significantly increased energy use and reduced swimming performance at warmer temperatures, potentially limiting successful recruitment [15]. Identifying this optimal temperature window for larval development is crucial for understanding the potential impacts of climate change, as rising water temperatures pose a threat to successful lobster reproduction [12,13]. Beyond the critical stage of larval development, water temperature also exerts a significant influence on various aspects of adult lobster behavior. Studies [16] suggest that temperature changes can impact lobster movement, distribution, and even catchability. The early life stages of lobster occur in the upper water column, making SST a relevant proxy for environmental conditions influencing survival and recruitment. Understanding these temperature-related behavioral changes, regardless of the specific temperature thresholds involved, is crucial for effective fishery management strategies in a changing climate. As water temperatures fluctuate due to climate change, lobster populations may alter their movement patterns and distribution, potentially impacting traditional fishing grounds. Additionally, temperature-induced behavioral changes could influence how readily lobsters interact with fishing gear, affecting catchability rates. Incorporating these behavioral considerations into fishery models will be essential for ensuring the sustainability of lobster populations in a warming ocean. While previous research has explored the relationship between ocean temperature and lobster populations in regions such as the Gulf of Maine and the northeastern United States, limited attention has been given to Prince Edward Island (PEI), which presents unique ecological, economic, and regulatory conditions. This study provides the first detailed analysis of lobster landings in PEI using a statistical modeling framework that incorporates both current and historical SST over an 8-year period, aligned with the species’ maturation timeline. By integrating long-term SST dynamics with fisheries management variables such as carapace length (CL), our approach contributes a regionally focused model that can inform adaptive strategies for climate-resilient fisheries management in Atlantic Canada. Therefore, any substantial change in water temperature may disrupt the lobster population’s distribution pattern and, thus, the stock in PEI. According to the Intergovernmental Panel on Climate Change (IPCC) Report [17], global SST will continue to increase until at least mid-century from historical emissions alone, and global warming of 1.5–2 °C above pre-industrial levels is likely to be exceeded during the 21st century unless there are sharp reductions in CO_2_ and other greenhouse gas emissions in the upcoming decades. The impacts of climate change on various systems (ecological and societal) would be significantly greater at 2 °C of global warming compared to 1.5 °C. These impacts, known as climatic impact drivers (CIDs), would become even more widespread and severe with further increases in global temperature. The ocean plays a vital role in regulating global climate, absorbing a significant portion of excess heat generated by human activities. Since 1970, oceans have absorbed an estimated 90% of this excess heat [18]. This warming trend poses a significant threat to marine ecosystems, particularly for organisms whose body temperature depends on their environment.

Marine organisms can respond to such changes by adapting rapidly to novel conditions, shifting their geographic ranges, altering behaviors (e.g., phenology, migration, etc.), or else experiencing local extirpations and inevitable extinctions where changes exceed tolerance thresholds. In the 20th century, observed responses to climate change include changes in species composition, abundance, and biomass production of ecosystems [18], which have ultimately driven declines in fisheries’ catches [18]. However, in some areas, changing ocean conditions have also contributed to the expansion of suitable habitats or increases in the abundance of some species in many ecosystems [18]. Anecdotal accounts from lobster fishermen in the state of Maine purport a reduced catch in recent years, while those on PEI are seeing an abundance of young lobster moving northward in search of cooler water due to climate change [19], the latter of which is supported by recent findings [20,21].

The objectives of the present study were to (1) examine the relationship between lobster landings and SST around PEI using Generalized linear Models, and (2) interpret these historical relationships in light of projected ocean warming under different emissions scenarios. This study specifically addresses the research question: How do historical SST patterns, including lag effects, influence lobster landings in Prince Edward Island? We hypothesize that lagged SST variables corresponding to key biological periods in the lobster life cycle are significant predictors of current landings. Like many ecological studies that look back at past data, this analysis has its limitations. We did not have access to bottom temperature data and we could not include factors like subsidies, changes in fishing gear, or whale-related area closures due to a lack of historical records.

## 2. Data and Methods

### 2.1. Data Collection

**Figure 1:**
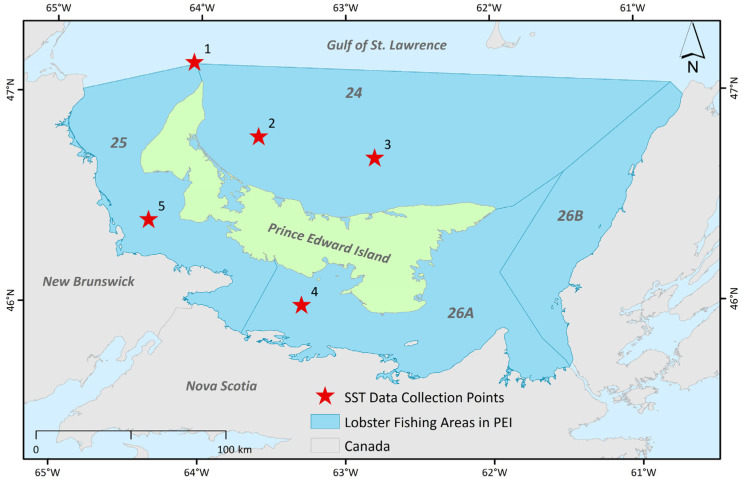
SST data collection points (red) in the water around PEI.

Monthly average SST around PEI was obtained from 1982 to 2021 from the Surface Temperature Group at the University of Reading, UK, at a grid resolution of 0.05° [22]. Data were collected from five location points within the LFAs in PEI (Figure 1). To generate the final SST dataset for each month, the SST values from representative locations were equally weighted, and monthly averages were calculated using the arithmetic mean. To understand potential long-term trends in SST around PEI, we utilized the Copernicus Climate Change Service (C3S) CMIP6 dataset. This dataset includes simulations for various emissions scenarios (SSP1-2.6, SSP2-4.5, and SSP5-8.5) covering the period from 2024 to 2099. While specific future projections are not presented in this study, these data allow us to explore how future climate change might impact PEI’s waters based on these scenarios. We employed the HadGEM3-GC3.1-LL (UK) climate model from CMIP6 due to its 1° resolution (providing detailed information on a grid of roughly 111 km × 111 km) and its ability to simulate realistic scenarios aligned with historical observations [23]. This information remains valuable for understanding potential future SST changes and their impact on PEI’s lobster fishery [24]. Thirty-two-year summaries of PEI lobster landing data from the period 1990 to 2021 were collected from the official website of Fisheries and Oceans Canada. Additionally, landing information from 2003 to 2022 for each LFA in PEI was collected from Fisheries and Oceans Canada’s office, as it was not available on the official website. Fishery license data for PEI (1990–2021) were obtained from Oceans Canada. The coordinates and shape files of LFAs in PEI were collected from Fisheries and Oceans Canada. LFA maps were generated using ArcGIS (v10.6.1), and the area of each zone was calculated using the “Calculate Geometry” function. Data analysis was performed using RStudio (version 2023.09.0+463).

### 2.2. Feature Selection

**Figure 2:**
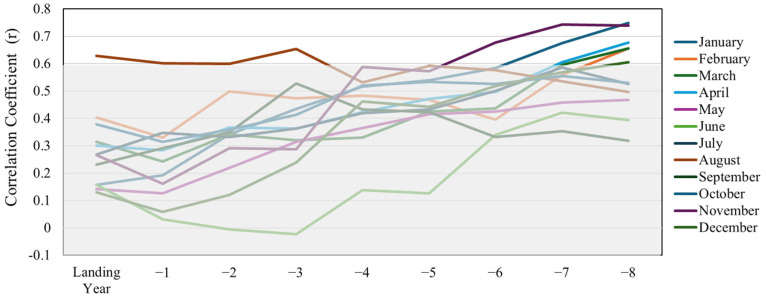
Selection of variables (SST °C) with correlation coefficient (r) values of 0.6 or above.

The Gulf of St. Lawrence has six lobster fishing zones, three of which coincide with the waters around PEI. These include zones 24 (ca. 19,090 km^2^), 26A (ca. 9498 km^2^), and 25 (ca. 7478 km^2^) (Figure 1). Landings have been previously found to differ between the three LFAs [25], but the present study found no difference between LFAs after standardizing landings by effort (i.e., number of fishing licenses). Therefore, we chose to use cumulative historical landings from all three LFAs of PEI (1990–2021) in our models. Landings per license were used as a proxy indicator of population trends because it does not directly measure population abundance. This metric may also be influenced by factors such as changes in fishing effort, gear efficiency, and market conditions. As lobsters require 5–8 years to grow to legal size, a model was built considering lobster’s exposure to the previous eight years of SST conditions [26,27,28]. In this way, our response variable (annual lobster landing in weight per license) was compared against explanatory variables that describe monthly and annual SST conditions from the preceding eight years [29]. We used a combination of the month and the year before the landing year to define individual SST variables. For example, aug_0 means SST in August of the same landing year, while may_5 means SST in May five years before the landing year. To reduce the dimensionality (i.e., number of variables) of our dataset, we examined Pearson’s correlations between our response and explanatory variables and set a cutoff correlation value of 0.6 for inclusion in our model. The correlation results were then verified by the Akaike Information Criterion (AIC) test before analysis. Figure 2 shows that SST variables with correlation coefficient (r) value lower than 0.6 fall in a grey area; therefore, those variables were discarded. To assess multicollinearity among the selected SST variables, a Variance Inflation Factor (VIF) analysis was conducted. All final model variables demonstrated VIF values below 5, indicating acceptable multicollinearity levels.

The final selected variables for the model are presented in Table 1. The variables aligned with lower AIC values, indicating better correlation. The explanatory variables selected for inclusion in our model include the following: average SST for August of the same year as the landing (denoted as aug_0); SSTs for August one, two, and three years before the landing year (denoted as aug_1, aug_2, and aug_3); SSTs for November six years before the landing year (denoted as nov_6); average SSTs (denoted as avg_7) of January, April, and November seven years (denoted as jan_7, aprl_7, nov_7) before the landing year; and average SSTs (denoted as avg_8) of January, February, March, April, November, and December eight years (denoted as jan_8, feb_8, mar_8, aprl_8, nov_8 and dec_8, respectively) before the landing year (Table 1). Variables with a cut-off value of 0.6 in the same year were averaged to reduce the number of total variables in the model (Table 2).

### 2.3. Model Selection and Statistical Analysis

Hypothesis testing was conducted using an alpha value of 0.05 to determine significance. The basic concept of the model was based on multiple linear regression, represented by the following equation:

Y = b_o_+ b_1_ × X_1_ + b_2_ × X_2_ + … + b_n_ × X_n_ + b_n+1_ × CL (1)

where ‘Y’ denotes historical lobster landing in weight (metric tonnes) per license in PEI; ‘X_1_, X_2_, …, X_n_’ denotes all the variables of Table 1; ‘b_1_, b_2_, …, b_n_’ denotes coefficients; ‘CL’ denotes the legal carapace length of lobster in PEI, and ‘b_o_’ denotes the intercept.

**Figure 3:**
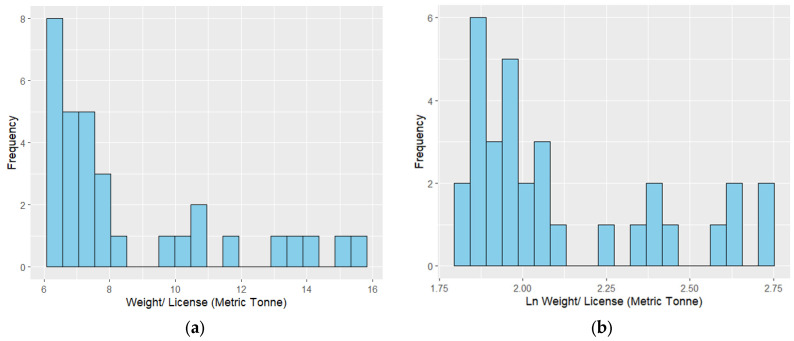
Histograms of (**a**) lobster landing and (**b**) the natural logarithm of lobster landing.

The response data (i.e., lobster landing in weight per license) showed a skewness of 1.184 Figure 3a. Usually, the Gamma distribution is appropriate for left-skewed data. However, in this study, the log-transformed response data shows a reduced skewness of 0.933 (Figure 3b).

**Figure 4:**
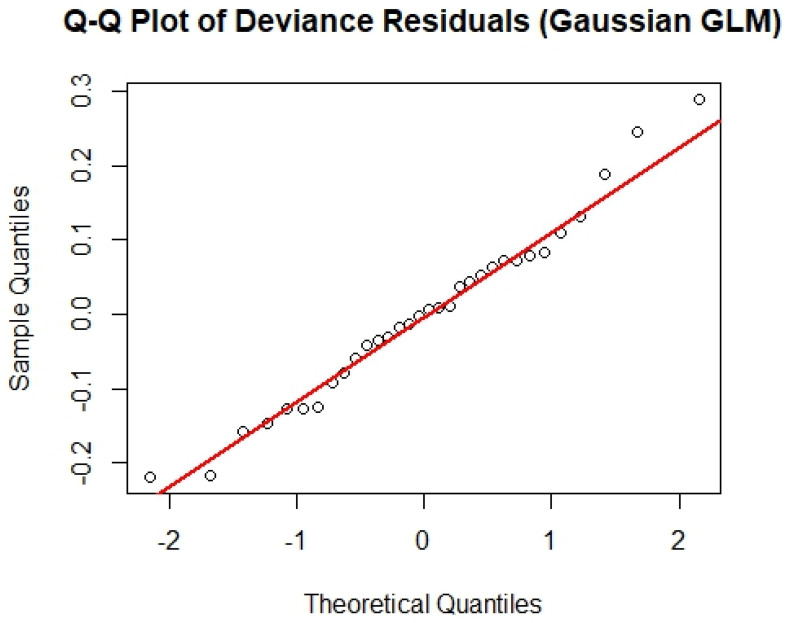
Q-Q plot of deviance residuals from the Gaussian model.

We chose a Gaussian GLM with a log-transformed response based on visual improvements in residuals and ease of interpretation. To evaluate the robustness of this choice, we also tested Gamma and Poisson (as a proxy for quasi-Poisson) GLMs using the same predictor set. The Gamma model produced a slightly lower AIC (−38.37) and deviance (0.088) compared to the Gaussian model (AIC = −33.14, deviance = 0.457), although the AIC difference was modest (ΔAIC~5). The Poisson model performed considerably worse. In the Q-Q plot (Figure 4) of deviance residuals from the Gaussian model, the red line represents the expected quantile from a standard normal distribution. The plot indicated approximate normality with only minor tail deviations. Residual dispersion was low (0.0169), supporting the adequacy of the log-Gaussian structure. Given these results and the model’s interpretability, we retained the Generalized Linear Model (GLM) with a Gaussian family as the final model. An AICc test within the GLM was performed primarily to check the fitness of the model. Variables with the highest sum of Akaike weights were considered the most “important predictors” of the response. The final historical model included eight explanatory variables (Table 1) using a GLM with the Gaussian family and identity link function. To avoid overfitting and following the 10:1 sample-to-variable rule, variables were eliminated in a stepwise fashion from the primary model according to *p* values until all remaining variables were significant (*p* < 0.05).

The inclusion of CL as a covariate in the model was based on the assumption that it could significantly influence catches and the recruitment dynamics of the fishery. Carapace length is a direct indicator of the size distribution of lobsters within the population, which is highly relevant to catch rates. A smaller minimum legal CL could lead to a higher potential catch since a larger proportion of the population becomes legally harvestable. This variability in CL thresholds across years and regions directly influences catchability and recruitment dynamics. Therefore, CL was included in the model for biological and regulatory relevance despite its lack of statistical significance (*p*-value = 0.706). We performed a sensitivity analysis to evaluate the importance of carapace length (CL). This involved comparing two GLMs: one including CL and one excluding it. The AIC for the model with CL was −33.14, while the AIC for the model without CL was slightly lower at −34.97. The small AIC difference (ΔAIC < 2) indicated similar model performance. Additionally, we tested interaction terms between CL and individual SST predictors. The interaction between aug_3 (SST lagged by 3 years) and CL showed the greatest model improvement (AIC = −42.48), suggesting that the influence of lagged SST on landings may be modulated by lobster size. While a full interaction model was also tested, it offered minimal improvement and increased complexity. Therefore, we focused on the most biologically meaningful single interaction. To explore the nonlinearity of CL, a second-order polynomial term (CL^2^) was also tested. While it significantly improved model fit, the main analysis retained the linear form of CL for consistency and interpretability. Except for CL, the Akaike weights result table obtained from the AICc test matched with the variables in the final model. Therefore, the final model contained four explanatory variables: aug_1, aug_3, avg_8, and CL.

By replacing the variables in the Equation (1), the final model equation is as follows:

ln (weight_L) = b_o_ + b_1_× (aug_1) + b_2_ × (aug_3) + b_3_ × (avg_8) + b_4_ × CL (2)

where ‘ln (weight_L)’ denotes the natural logarithm of lobster landing in weight (metric tonnes) per license in PEI; ‘aug_1’ denotes SSTs for August one year before the landing year; ‘aug_3’ denotes SSTs for August three years before the landing year; and ‘avg_8’ denotes average SSTs of January, February, March, April, November, and December eight years before the landing year. The final GLM model was then used to predict future annual lobster landings around PEI between 2032 and 2099.

## 3. Results

### 3.1. Lobster Fishery in PEI

**Figure 5:**
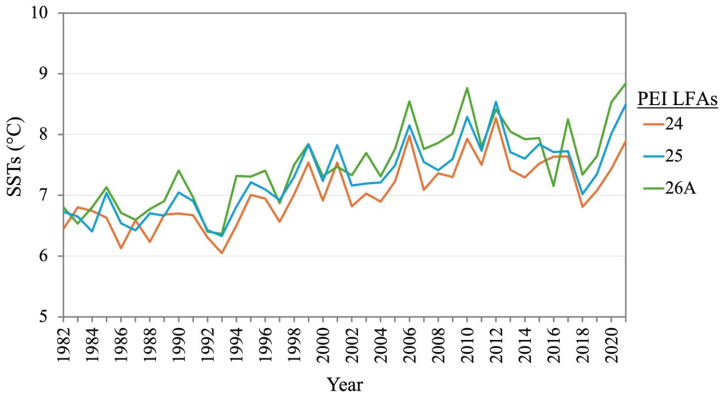
Annual SST trend in three LFAs from 1982 to 2021 in PEI.

**Figure 6:**
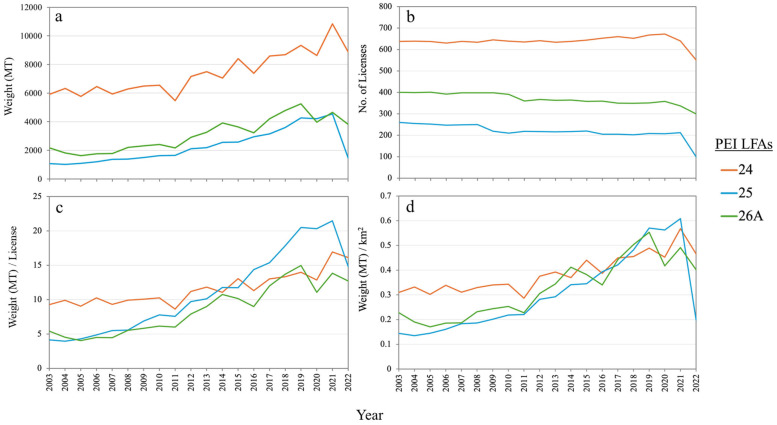
Annual lobster landings (**a**), number of licenses (**b**), landings per license (**c**), and landings per km^2^ (**d**) in the three LFAs around PEI between 2003 and 2022.

SST around PEI has warmed at an average rate of 0.1 °C/decade over the past four decades [30]. Among the three LFAs around PEI, LFA 24 is slightly cooler, while 26A is slightly warmer than the others (Figure 5). The most successful of the LFAs in terms of annual landings happens to be the largest in the area (LFA 24), but when standardized by effort (number of licenses), the smaller LFA 25 has been relatively the most successful over the past decade (Figure 6c), with fewer number of licenses compared to the other two LFAs. LFA 25 had the highest production per license when it allowed licenses between 204 and 212 until it further decreased to 100 in 2022 (Figure 6b,c). Landing per square kilometer showed an almost similar trend in the three LFAs (Figure 6d).

### 3.2. AICc and Generalized Linear Model (GLM) Result

Based on the AICc test, the lowest AICc value indicates the best model. We found model no. 171, 169, 44, 172, and 48 had the lowest AICc values and were not significantly distinguishable (ΔAICc < 2) (Table 3), suggesting that more than one model could explain the data well. Although we chose the model with the lowest AICc for interpretation, we recognize that other models were also supported. We chose to focus on a single model for simplicity, but future research may benefit from using model averaging or ensemble methods to improve robustness and capture uncertainty in variable selection.

Table 4 shows that the variables with the highest sum of weights were avg_8, aug_3, and aug_1. The table adds Akaike weights of all models that include a given predictor in the 95% confidence set. N containing models refers to the number of models in the 95% confidence set that included a given variable.

Our GLM also revealed that “aug_1”, “aug_3”, and “avg_8” variables were significant predictors of lobster landings (*p* < 0.01) (Table 3). The “avg_8” variable had the strongest (positive) relationship with annual landings (*p* < 0.0001) (Table 5), such that for every ºC increase in “avg_8” (i.e., average SST of January, February, March, April, May, November, and December eight years before the landing year), annual landings are expected to increase by 0.36 metric tonnes with all else being constant. This variable likely reflects the long-term biological linkage between early life stage environmental conditions and future recruitment. Since lobsters in PEI typically take 5–8 years to reach legal harvest size, SST eight years prior may represent the ocean conditions experienced during early benthic or juvenile stages, ultimately influencing the size and survival of the harvested cohort. However, “CL” had a weaker effect and was statistically less significant than the other predictors. This means SST has a larger impact on lobster landing than restricting carapace length as a management measure. The result still shows that restricting lobster harvest by increasing the one-millimeter legal carapace length will support increased landing because it will help protect the stock.

Ideally, in a model, the center of the deviation residuals should be zero. As shown in Table 6, the median number (−0.003) is near zero, which is a good indicator. Since the lower deviance represents a better fit, the residual deviance in the model’s output indicates that the model and the observed data fit at a reasonable level (Table 7). The table also indicates that the model is a good fit for the data because its residual (model) deviance is smaller than the null (intercept-only) deviance.

**Figure 7:**
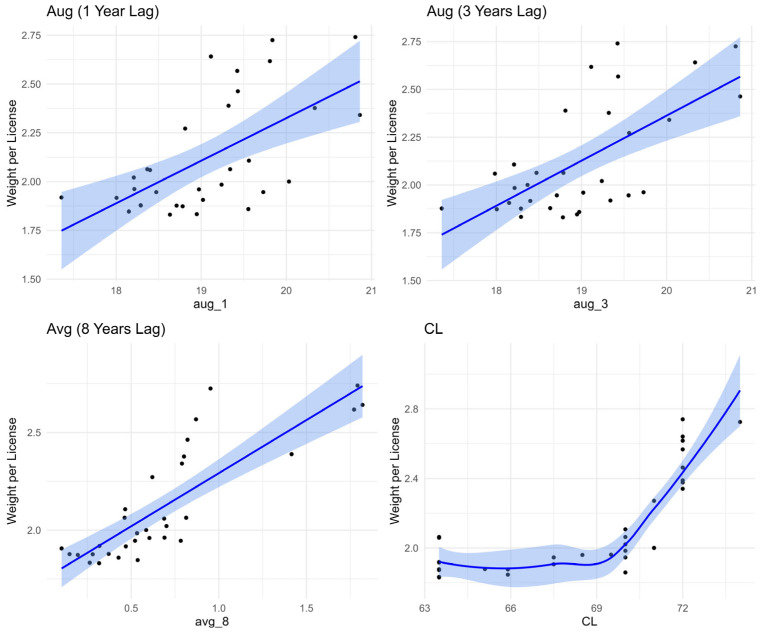
Distribution plot of the predictor variables against landing per license, with observed data (black dots), regression lines (blue lines), and associated 95% confidence intervals (blue bands).

In Figure 7, the SST of August one year before the landing year (aug_1) and the SST of August three years before the landing year (aug_3) show better linear distribution than the average SST of January, February, March, April, November, and December (avg_8) eight years before the landing year. Unlike all SST variables modeled, which show relatively linear relationships with lobster landings per license, CL had a positive non-linear relationship similar to an exponential growth curve (Figure 7). Although the final model used a linear term for CL, we found that including a quadratic term significantly improved model fit and confirmed a nonlinear effect. This result suggests that lobster landings increase with CL at an accelerating rate, which may reflect biological or fishery dynamics.

**Figure 8:**
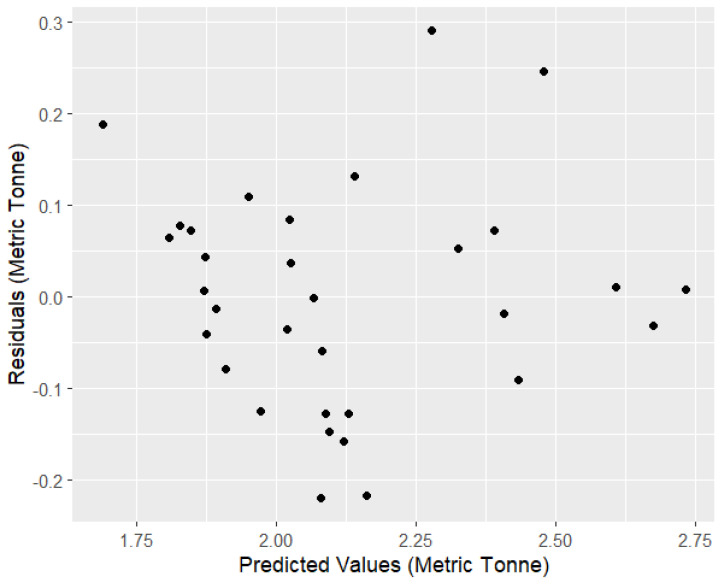
Residuals vs. predicted value of the model.

**Figure 9:**
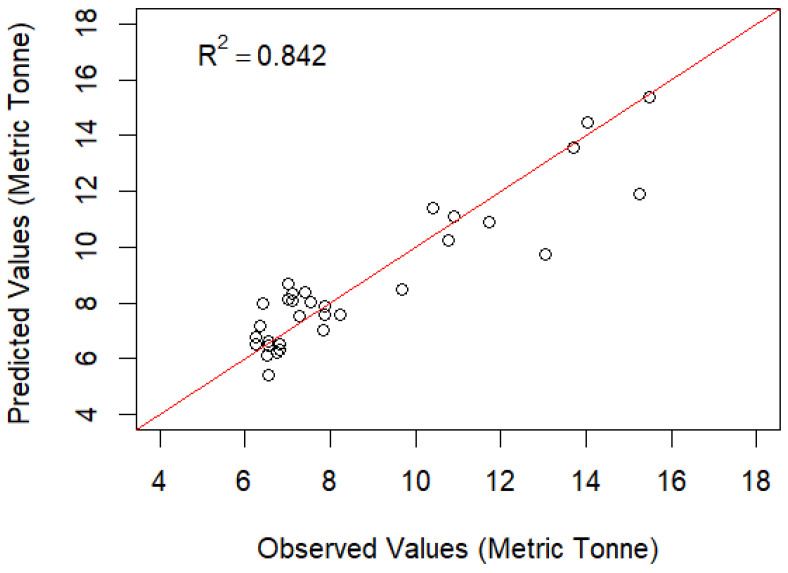
Difference between observed and predicted values from 1990 to 2021.

**Figure 10:**
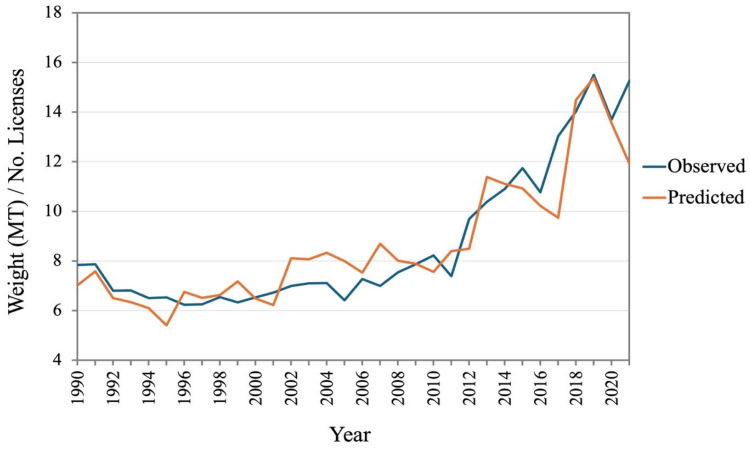
Timeline of observed vs. predicted landing per license in PEI LFAs.

The residual dispersion along the *y*-axis shows the errors (Figure 8). A residual distribution near zero suggests that the model is producing reliable predictions. On the other hand, if the dispersion is not even or if it shows any patterns, it indicates that the model has limitations or assumptions may not hold. As shown in Figure 8, the distribution of the residuals is randomly scattered, therefore following a standard normal distribution. This means the model is reasonably fitted. In Figure 8, the values on one side of the line represent the actual values, and those on the other represent the predicted values. Data points that fall precisely on the line have predicted values that match the actual values exactly. In Figure 9, the red line represents the 1:1 relationship where predicted values exactly match observed values. Values above and below the line represent overestimations and underestimations of the predicted value. However, when the historical predicted values from the model and observed values are plotted in a timeline (Figure 10), it shows that the prediction values closely follow the observed values.

To evaluate how well the final GLM model predicts lobster landings, we calculated both in-sample and cross-validated accuracy metrics. The in-sample Root Mean Squared Error (RMSE) and Mean Absolute Error (MAE) were 0.1195 and 0.0935, respectively. These low values suggest that the model fits the data well, with small average differences between observed and predicted values. To further assess model stability, we performed 5-fold cross-validation. The cross-validated RMSE (0.138) and MAE (0.108) were only slightly higher than the in-sample values (0.1195 and 0.0935, respectively), suggesting that the model performs consistently and is not overfitting to the training data. These results, along with residual diagnostics (Q–Q plots in Figure 4), support the reliability of the AICc-selected model despite the limited sample size (*n* = 32 years).

## 4. Discussions

The results of this study build upon and extend the existing literature on the impacts of ocean temperature on lobster populations by offering a region-specific, temporally integrated analysis focused on PEI. Our model incorporates a biologically informed 8-year SST exposure window to reflect the full maturation period of lobsters. Additionally, the inclusion of regulatory parameters such as carapace length (CL) introduces a management dimension that has not been widely considered in similar models. These elements offer novel insights into how both environmental and regulatory variables jointly influence landings, providing a valuable foundation for local adaptation strategies in climate-sensitive fisheries.

In this study, SST was utilized as a proxy for ocean floor temperature, which is crucial for understanding lobster life cycles. While we aimed to investigate the potential impacts on lobster populations across Lobster Fishing Areas (LFAs) in Prince Edward Island (PEI), time series data for bottom temperatures are often unavailable. Consequently, we relied on SST, recognizing that while it generally correlates with water depth, variations across LFAs may lead to discrepancies in the specific temperature conditions experienced by lobsters. This limitation should be acknowledged when interpreting the results in relation to spatial variations within the fishery. Additionally, it is important to note that lobster larvae inhabit the upper water column before settling on the ocean floor, indicating that their life cycle is not entirely independent of SST. Landings per license were used as a proxy for relative lobster abundance due to the lack of consistent, long-term fishery-independent data. While this metric offers a practical and standardized measure over time, it does not directly reflect true population size and may be influenced by changes in effort, fisher behavior, market, quota, gear efficiency, or other factors [31]. In PEI, the number of fishing days fluctuates annually due to weather conditions, and restrictions related to the presence of endangered species, such as North Atlantic whales, can further limit fishing opportunities. This reduction in available fishing days may impact landings, especially if fishermen start later in the season. On the other hand, access to credits and government subsidies for lobster fishermen also influences harvesting. The interplay of increased SST, changes in the total number of fishing licenses, and stringent management regulations (such as carapace length requirements) may also have influenced annual landings in PEI in recent years. Therefore, the results between SST and landings should be interpreted with caution, and they should be interpreted as correlations, not direct causation. Future studies should consider integrating the above-mentioned factors where available. A lack of sufficient data prevented us from exploring these factors, marking another limitation of the study. The current study observed that the SST around PEI has historically increased over time. According to the present climate scenario, this trend is expected to continue in the coming decades [18]. An important consequence of climate change that might have effects on lobsters in the future is acidification. Ocean acidification accelerates the chemical reactions that reduce the quantity of calcium carbonate in salt water, impacting calcifying organisms’ ability to form shells [32]. Reducing calcium carbonate availability weakens the shells and exoskeletons of crustaceans (e.g., lobsters) and makes them more vulnerable to dissolution [33]. The process also lowers dissolved oxygen levels in the water and affects the movement of predators. Besides acidification, factors like nutrition impact lobster molting and shell hardness [9]. Additionally, repeated handling of egg-bearing females by fishermen can cause egg loss and affect incubation [2]. Any stress (e.g., temperature above 20 °C) [34] that disrupts spawning, egg attachment, incubation, hatching, larval growth, sexual maturity, and movement can negatively affect lobster recruitment to later life stages [9]. Therefore, changes in temperature, acidity, disease spread, food availability, biomass, and salinity(e.g., evaporation and ice melting), can be contributing factors to population shifts [14,35,36]. Unfortunately, these important factors were not included in our models due to a lack of available data.

Our results support those from previous studies that explain how temperature plays a crucial factor in early maturation [37], molting [25], embryonic development [37,38], hatching [39,40,41], larval development [20,42,43,44,45], and harvest [21] of lobster. Recent studies further support the positive correlation between warm ocean temperatures and lobster harvest [46]. The historical model output reflected the relationship of SST in different months and years on the life stages of lobster. For example, mating, molting, spawning, and hatching of mature American lobsters usually occur in warmer months when the water temperature is relatively higher [9,38,47,48]. In this study, the coefficient values for August SST one and three years before the landing year support the idea that relatively older lobsters respond to warmer temperatures for mating, molting, spawning, and hatching. Similarly, the strong effect of the average SST of January, February, March, April, November, and December eight years before the landing year (avg_8) likely captures environmental conditions during key early juvenile phases. Given that lobsters require up to 5–8 years to reach harvestable size [26], SST eight years prior reasonably influenced the survival, growth, or recruitment probability of the individuals harvested in the current year. These findings align with previous research showing that elevated SST can impair larval migration and survival due to increased energetic demands, thereby influencing recruitment success [15].

Regional ocean warming presents a complex picture for PEI lobster populations. Recent studies approve the impact of warming waters on lobster behavior, physiology, and range, as wells as the challenges of the fisheries sector, including the potential loss of habitat suitability [49]. In PEI, a decline in stock is a potential consequence, but other scenarios may also emerge. Additionally, the specific impact of warming on lobster populations will likely be influenced by a multitude of interacting climate change factors beyond temperature alone. These factors include changes in ocean acidification, lower oxygen levels, shell disease, and prey availability, all of which can have significant effects on lobster physiology, behavior, and overall population health. Understanding how these various stressors interact with rising temperatures will be crucial for predicting the future of the PEI lobster fishery under a changing climate. Model projections suggest that by 2098, SST around PEI will reach only 15.76 °C under the highest emissions scenario (SSP5-8.5). Previous studies have indicated that juvenile lobsters and larvae may experience physiological stress, lower molting success, and reduced survival at temperatures exceeding 18–20 °C [9,50,51,52]. While our model focused primarily on thermal conditions, other key stressors such as ocean acidification, hypoxia, and shell disease were out of scope and not included due to data limitations. It is important to consider these elements because they affect lobster physiology and survival, and therefore should be included in future projection models for PEI lobster when long-term data become available. The interpretation from this study indicates that the future impact of rising temperatures on lobsters remains uncertain, considering the combined effects of ocean acidification, disease, and other ecological factors. However, it is reasonable to state that if proper climate actions are taken to limit emissions within the SSP1-2.6 and SSP2-4.5 scenarios, the SST around PEI will not reach 15.76 °C. The fate of fisheries requires understanding the local interaction between life stage-specific biological thresholds and oceanographic processes on a finer scale [20].

The lobster industry must prioritize adaptive measures that align with projected changes in ocean temperatures and ecosystem dynamics [53]. These may include revising fishing quotas, diversifying target species, and enhancing habitat restoration efforts to mitigate the impact of warming on lobster populations. Furthermore, the development of predictive models and monitoring tools to track changes in lobster distribution, growth, and breeding patterns can provide valuable insights for sustainable resource management in PEI. By integrating adaptive management with long-term greenhouse gas (GHG) reduction efforts, the lobster industry can potentially address both immediate and future challenges. A dual approach ensures the industry remains resilient in the face of ongoing climate stressors while contributing to the broader goal of mitigating the impacts of global warming. However, these strategies are not based directly on the results of our model. Instead, they are drawn from broader discussions in the literature and are intended as general ideas to consider in future planning. These examples are meant to highlight possible ways the industry could adapt, rather than recommendations supported by our analysis. Our results suggest a positive correlation between warmer ocean temperatures and lobster landings; however, this relationship should be interpreted cautiously, as it may also reflect unmeasured factors such as changes in fishing behavior, market dynamics, or regulatory enforcement. These data gaps and limitations underscore the need for comprehensive future research that includes additional data on catchability, population abundance, and other relevant factors, leading to a more robust understanding of how climate change and SST variations might influence PEI lobster fishery. Climate change presents a complex challenge, and several other environmental factors beyond temperature are likely to influence lobster populations in Prince Edward Island. Ocean acidification, caused by rising atmospheric CO_2_, may pose a greater threat than rising temperatures, as it can disrupt lobster shell development and survival. Climate change could also facilitate the introduction of new diseases or predators, further jeopardizing lobster populations. Additionally, shifts in prey distribution and abundance due to climate variations might impact food sources and, ultimately, lobster health. Given these complexities, accurately forecasting future lobster stock dynamics remains a challenge. However, our findings on temperature sensitivity offer valuable insights into potential vulnerabilities. While rising temperatures under different emissions scenarios might initially benefit lobster populations in areas with suitable habitats, the long-term consequences of combined climate stressors require further investigation through more comprehensive models that incorporate these additional environmental factors.

## 5. Conclusions

The future of lobster fisheries in PEI in the context of climate change is a complex and evolving issue that involves both opportunities and challenges. This study provides empirical evidence of a statistically significant relationship between sea surface temperature (SST) and lobster landings in Prince Edward Island (PEI), using a Generalized Linear Model (GLM). Specifically, the model demonstrated significant lagged effects of SST, particularly for the months of August one and three years prior to landing, and the average SST eight years prior, corresponding with key biological periods in the lobster life cycle. These findings suggest that past ocean temperatures have a quantifiable influence on current lobster landings, providing important insights for understanding how future ocean warming scenarios might impact the PEI lobster fishery. While the results highlight significant trends, and the model captured climate-related signals, its explanatory power is limited by data constraints and the absence of important policies and biological and environmental variables, indicating the need for more comprehensive analyses that incorporate additional key factors to fully assess long-term sustainability.

### Recommendations for Sustainable Lobster Fisheries

While this study focused primarily on the relationship between SST and lobster landings, we acknowledge the importance of exploring broader factors influencing fishery sustainability. Based on the empirical findings, we propose the following considerations for future research and potential management strategies:

1. Conduct expanded studies incorporating biological factors, such as lobster recruitment dynamics, disease prevalence, and predator-prey relationships.
2. Evaluate socio-economic factors influencing fishery outcomes, including access to markets and policy regulations.
3. Develop predictive models integrating environmental, biological, and economic variables for robust climate-resilient fishery planning.
4. Encourage collaboration between researchers, policymakers, and stakeholders to develop adaptive management strategies informed by ongoing scientific research.
5. Consider pilot initiatives for habitat restoration and diversification of target species to enhance ecosystem resilience.

These recommendations are informed by our study’s findings but are intended as directions for future exploration rather than immediate policy prescriptions.

## Figures and Tables

**Table 1:** The final selected variables for the model.

Variable	Correlation Coefficient (Landing)	Correlation Coefficient (Landing per License)	AICc
avg_8	0.75	0.839	−21.19
avg_7	0.75	0.796	−15.01
CL	0.71	0.721	−8.48
nov_6	0.67	0.677	−3.86
aug_3	0.65	0.652	−0.29
aug_0	0.63	0.628	0.50
aug_2	0.59	0.600	1.93
aug_1	0.59	0.600	2.81

**Table 2:** Variables with correlation coefficient (r) value greater than or equal to 0.6.

Variable	Correlation Coefficient (Landing)	Correlation Coefficient (Landing per License)	AICc
avg_8	0.75	0.839	−21.19
avg_7	0.75	0.796	−15.01
nov_7	0.74	0.742	−10.22
nov_8	0.74	0.739	−8.91
CL	0.71	0.721	−8.48
jan_8	0.76	0.749	−8.36
nov_6	0.67	0.677	−3.86
jan_7	0.67	0.674	−2.24
apr_8	0.68	0.677	−0.54
aug_3	0.65	0.652	−0.29
feb_8	0.66	0.655	0.25
aug_0	0.63	0.628	0.50
aug_2	0.59	0.600	1.93
dec_8	0.61	0.605	2.08
mar_8	0.65	0.655	2.13
aug_1	0.59	0.600	2.81
apr_7	0.60	0.605	3.84

**Table 3:** Output of the AIC test.

Model #	Intercept	aug_0	aug_1	aug_2	aug_3	avg_7	avg_8	CL	nov_6	R^2^	AICc	Akaike Weight
171	−1.543		0.0657		0.0822		0.333		0.0919	0.8566	−35.9	0.194
169	−0.503				0.0811		0.3606		0.1238	0.8343	−34.3	0.088
44	−2.48	0.0634	0.0870		0.0789		0.3378			0.8464	−33.7	0.065
172	−1.943	0.0337	0.0669		0.0754		0.3236		0.0724	0.8611	−33.6	0.062
48	−3.369	0.0659	0.0812	0.0535	0.0773		0.2833			0.8599	−33.3	0.054
175	−1.964		0.0666	0.0241	0.0835		0.313		0.0821	0.859	−33.1	0.049
235	−1.502		0.0712		0.0883		0.3414	−0.0044	0.0970	0.8574	−32.7	0.041
43	−1.852		0.0973		0.0981		0.3702			0.8256	−32.7	0.039
170	−0.8477	0.03055			0.0749		0.3526		0.1067	0.8379	−32	0.027
47	−2.658		0.0923	0.0500	0.0973		0.3205			0.8375	−31.9	0.026
163	−0.1374		0.0643				0.3951		0.1126	0.8206	−31.8	0.025
173	−0.8547			0.0208	0.0822		0.3437		0.1157	0.8361	−31.6	0.023
233	−0.6446				0.0744		0.3487	0.0048	0.1152	0.8354	−31.5	0.021
176	−2.692	0.0438	0.06873	0.0359	0.0753		0.291		0.0519	0.866	−31.1	0.018
161	0.8618						0.4213		0.1435	0.7993	−31	0.017
164	−0.9528	0.0532	0.06643				0.3722		0.0790	0.8324	−30.9	0.016
112	−3.653	0.0780	0.09726	0.0676	0.0921		0.2912	−0.0118		0.8647	−30.8	0.016
36	−1.491	0.0868	0.08842				0.3902			0.8149	−30.7	0.015
108	−2.491	0.0659	0.09094		0.0823		0.3427	−0.0026		0.8467	−30.4	0.013
236	−1.932	0.0380	0.07557		0.0838		0.3353	−0.0067	0.0777	0.8629	−30.4	0.013
40	−2.445	0.0889	0.08226	0.0561			0.332			0.8297	−30.4	0.012
225	0.1357						0.373	0.0139	0.1141	0.8109	−30.1	0.011
239	−2.009		0.07583	0.0304	0.0937		0.3214	−0.0071	0.0878	0.8609	−29.9	0.01
162	0.1269	0.0499					0.4006		0.113	0.8097	−29.9	0.01
107	−1.876		0.08995		0.0910		0.3595	0.0044		0.8266	−29.8	0.009

**Table 4:** Sum of Akaike weights per variables obtained from the AIC test.

	avg_8	aug_3	aug_1	nov_6	aug_0	aug_2	CL
The sum of weights:	1	0.85	0.75	0.72	0.39	0.27	0.18
N containing models:	40	25	25	27	22	17	16

**Table 5:** Output of the GLM historical model.

	Estimate	SE	t-Value	*p*-Value
Intercept	−1.876	0.832	−2.255	0.033 *
aug_1	0.090	0.038	2.392	0.024 *
aug_3	0.091	0.039	2.347	0.027 *
avg_8	0.360	0.071	5.033	2.78 × 10^−5^ **
CL	0.004	0.012	0.381	0.706

Note: ‘*’ indicates significance level of 0.01; ‘**’ indicates significance level less than 0.01.

**Table 6:** Deviance residuals of the model.

Min	1Q	Median	3Q	Max
−0.220	−0.082	−0.003	0.072	0.290

**Table 7:** Deviance of the model.

Parameters	Values
Null deviance (metric tonne per license)	2.635
Residual deviance (metric tonne per license)	0.457

## Data Availability

The original contributions presented in the study are included in the article, further inquiries can be directed to the corresponding author.

## References

[1] Coulter M., PNI Atlantic (2018). P.E.I. lobster fishermen can expect good shore prices this year. The Guardian.

[2] Fisheries and Oceans Canada (2022). Information Brochure on the American Lobster. https://catalogue.ogsl.ca/dataset/ca-cioos_e9d86c62-9cbb-4cd4-83a2-cff29e626090?local=en.

[3] P. E. I. Statistics Bureau (2021). Province of Prince Edward Island Forty-Seventh Annual Statistical Review 2020.

[4] Department of Fisheries and Aquaculture (2009). Annual Report 2007–2008.

[5] Lawton P. (1995). Post larval, Juvenile, Adolescent, and Adult Ecology. Biology of the Lobster Homarus americanus.

[6] Pringle J.D., Burke D.L. (1993). The Canadian Lobster Fishery and Its Management, with Emphasis on the Scotian Shelf and the Gulf of Maine. Perspect. Can. Mar. Fish. Manag. Can. Bull. Fish. Aquat. Sci..

[7] Cooper R.A., Uzmann J.R. (1971). Migrations and Growth of Deep-Sea Lobsters, *Homarus americanus*. Science.

[8] Cobb S.J. (1976). The American Lobster: The Biology of Homarus americanus.

[9] Aiken D.E., Waddy S.L. (1986). Environmental Influence on Recruitment of the American Lobster *Homarus americanus*: A Perspective. Can. J. Fish. Aquat. Sci..

[10] Cobb J.S., Phillips B.F. (1980). The Biology and Management of Lobsters: Physiology and Behavior.

[11] MacKenzie C., Moring J.R. (1985). Species Profiles: Life Histories and Environmental Requirements of Coastal Fishes and Invertebrates (North Atlantic): American Lobster.

[12] Quinn B.K., Sainte-Marie B., Rochette R., Ouellet P. (2013). Effect of Temperature on Development Rate of Larvae from Cold-Water American Lobster (*Homarus americanus*). J. Crustac. Biol..

[13] Quinn B.K. (2017). Threshold Temperatures for Performance and Survival of American Lobster Larvae: A Review of Current Knowledge and Implications to Modeling Impacts of Climate Change. Fish. Res..

[14] Wahle R.A., Dellinger L., Olszewski S., Jekielek P. (2015). American Lobster Nurseries of Southern New England Receding in the Face of Climate Change. ICES J. Mar. Sci..

[15] García-Echauri L.L., Liggins G., Cetina-Heredia P., Roughan M., Coleman M.A., Jeffs A. (2020). Future Ocean Temperature Impacting the Survival Prospects of Post-Larval Spiny Lobsters. Mar. Environ. Res..

[16] Drinkwater K.F., Tremblay M.J., Comeau M. (2006). The Influence of Wind and Temperature on the Catch Rate of the American Lobster (*Homarus americanus*) during Spring Fisheries off Eastern Canada. Fish. Oceanogr..

[17] Intergovernmental Panel on Climate Change (IPCC) (2023). Climate Change 2021—The Physical Science Basis: Working Group I Contribution to the Sixth Assessment Report of the Intergovernmental Panel on Climate Change.

[18] Intergovernmental Panel on Climate Change (IPCC) (2022). The Ocean and Cryosphere in a Changing Climate: Special Report of the Intergovernmental Panel on Climate Change.

[19] Berardelli J. (2021). Maine’s lobster industry is thriving thanks to climate change—But it won’t last if the waters continue to warm. CBS NEWS.

[20] Goode A.G., Brady D.C., Steneck R.S., Wahle R.A. (2019). The Brighter Side of Climate Change: How Local Oceanography Amplified a Lobster Boom in the Gulf of Maine. Glob. Change Biol..

[21] Oppenheim N.G., Wahle R.A., Brady D.C., Goode A.G., Pershing A.J. (2019). The Cresting Wave: Larval Settlement and Ocean Temperatures Predict Change in the American Lobster Harvest. Ecol. Appl..

[22] Merchant C.J., Embury O., Bulgin C.E., Block T., Corlett G.K., Fiedler E., Good S.A., Mittaz J., Rayner N.A., Berry D. (2019). Satellite-Based Time-Series of Sea-Surface Temperature since 1981 for Climate Applications. Sci. Data.

[23] Andrews M.B., Ridley J.K., Wood R.A., Andrews T., Blockley E.W., Booth B., Burke E., Dittus A.J., Florek P., Gray L.J. (2020). Historical Simulations with HadGEM3-GC3.1 for CMIP6. J. Adv. Model. Earth Syst..

[24] Siedlecki S.A., Salisbury J., Gledhill D.K., Bastidas C., Meseck S., McGarry K., Hunt C.W., Alexander M., Lavoie D., Wang Z.A. (2021). Projecting Ocean Acidification Impacts for the Gulf of Maine to 2050: New Tools and Expectations. Elem. Sci. Anthr..

[25] Thakur K.K., Revie C., Stryhn H., Tibbetts S.S., Lavallée J., Vanderstichel R. (2017). Risk Factors Associated with Soft-Shelled Lobsters (*Homarus americanus*) in Southwestern Nova Scotia, Canada. FACETS.

[26] Wilder D.G. (1953). The Growth Rate of the American Lobster (*Homarus americanus*). J. Fish. Res. Board Can..

[27] Tremblay M.J., Eagles M.D. (1996). Recent Trends in the Lobster Fishery off Eastern Cape Breton (LFA’s 27-30): Catch Rate and Exploitation. DFO Atlantic Fisheries Research Document 96/141. https://waves-vagues.dfo-mpo.gc.ca/library-bibliotheque/211172.pdf.

[28] NOAA Fisheries *Fun Facts About Luscious Lobsters*. NOAA Fisheries. https://www.fisheries.noaa.gov/national/outreach-and-education/fun-facts-about-luscious-lobsters#how-does-a-lobster-grow.

[29] Huntsberger C.J., Kilada R., Ambrose Jr W.G., Wahle R.A. (2020). Age-at-Size Relationships of the American Lobster (*Homarus americanus*) from Three Contrasting Thermal Regimes Using Gastric Mill Band Counts as a Direct Aging Technique. Can. J. Fish. Aquat. Sci..

[30] Maqsood J., Wang X., Farooque A.A., Nawaz R.A. (2024). Future Projections of Temperature-Related Indices in Prince Edward Island Using Ensemble Average of Three CMIP6 Models. Sci. Rep..

[31] Gardner C., Larkin S., Seijo J.C., Phillips B.F. (2013). Systems to Maximize Economic Benefits in Lobster Fisheries. Lobsters: Biology, Management, Aquaculture and Fisheries.

[32] Waterman M. (2018). Ocean Acidification May Affect Lobster Molt, Reproduction. Maine Lobstermen’s Community Alliance.

[33] Intergovernmental Panel on Climate Change (IPCC) (2023). Climate Change 2022—Impacts, Adaptation and Vulnerability: Working Group II Contribution to the Sixth Assessment Report of the Intergovernmental Panel on Climate Change.

[34] McLeese D.W., Wilder D.G. (1958). The Activity and Catchability of the Lobster (*Homarus americanus*) in Relation to Temperature. J. Fish. Board Can..

[35] Greenhalgh E. *Climate & Lobsters*. Climate.gov. https://www.climate.gov/news-features/climate-and/climate-lobsters.

[36] Carloni J.T., Wahle R., Geoghegan P., Bjorkstedt E. (2018). Bridging the Spawner-Recruit Disconnect: Trends in American Lobster Recruitment Linked to the Pelagic Food Web. Bull. Mar. Sci..

[37] Templeman W. (1936). Local Differences in the Life History of the Lobster (*Homarus americanus*) on the Coast of the Maritime Provinces of Canada. J. Biol. Board Can..

[38] Perkins H.C. (1972). Developmental Rates at Various Temperatures of Embryos of the Northern Lobster (*Homarus americanus* Milne-Edwards). U.S. Dep. Commer. Fish. Bull..

[39] Harding G.C., Vass W.P., Drinkwter K.F. (1982). Aspects of Larval American Lobster (*Homarus americanus*) Ecology in St. Georges Bay, Nova Scotia. Can. J. Fish. Aquat. Sci..

[40] Harding G.C., Drinkwater K.F., Vass W.P. (1983). Factors Influencing the Size of American Lobster (*Homarus americanus*) Stocks along the Atlantic Coast of Nova Scotia, Gulf of St. Lawrence, and Gulf of Maine: A New Synthesis. Can. J. Fish. Aquat. Sci..

[41] Hughes J.T., Matthiessen G.C. (1962). Observations on the Biology of the American Lobster, *Homarus americanus*. Limnol. Oceanogr..

[42] Huntsman A.G. (1923). Natural Lobster Breeding.

[43] Caddy J.F. (1976). The Influence of Variations in the Seasonal Temperature Regime on Survival of Larval Stages of the American Lobster (Homarus americanus) in the Southern Gulf of St. Lawrence [Canada].

[44] MacKenzie B.R. (1988). Assessment of Temperature Effects on Interrelationships between Stage Durations, Mortality, and Growth in Laboratory-Reared *Homarus americanus* Milne Edwards Larvae. J. Exp. Mar. Biol. Ecol..

[45] Ennis G.P., Factor J.R. (1995). Chapter 3—Larval and Postlarval Ecology. Biology of the Lobster.

[46] Wright D., Liu Y. (2024). Assessing the Impact of Environmental Variability on Harvest in a Heterogeneous Fishery: A Case Study of the Canadian Lobster Fishery. J. Environ. Econ. Policy.

[47] Perkins H.C. (1971). Egg Loss during Incubation from Offshore Northern Lobsters (Decapoda: Homaridae). Fish. Bull..

[48] Templeman W. (1936). The Influence of Temperature, Salinity, Light and Food Conditions on the Survival and Growth of the Larvae of the Lobster (*Homarus americanus*). J. Biol. Board Can..

[49] Watson W.H., Jury S.H., Gutzler B.C., Lippmann T.C., Choi J.-G., Carloni J.T., Goldstein J.S. (2023). How Might Climate Change Affect American Lobsters?. Front. Young Minds.

[50] Aiken D.E., Waddy S.L. (1976). Controlling Growth and Reproduction in the American Lobster. Proc. Annu. Meet.—World Maric. Soc..

[51] Quinn B.K., Rochette R. (2015). Potential Effect of Variation in Water Temperature on Development Time of American Lobster Larvae. ICES J. Mar. Sci..

[52] Templeman W. (1933). The Effect of Environmental Conditions on the Survival of Lobster Larvae.

[53] Mills K.E., Pershing A.J., Brown C.J., Chen Y., Chiang F.-S., Holland D.S., Lehuta S., Nye J.A., Sun J.C., Thomas A.C. (2013). Fisheries Management in a Changing Climate: Lessons from the 2012 Ocean Heat Wave in the Northwest Atlantic. Oceanography.

